# Identification of highly connected and differentially expressed gene subnetworks in metastasizing endometrial cancer

**DOI:** 10.1371/journal.pone.0206665

**Published:** 2018-11-01

**Authors:** Kanthida Kusonmano, Mari K. Halle, Elisabeth Wik, Erling A. Hoivik, Camilla Krakstad, Karen K. Mauland, Ingvild L. Tangen, Anna Berg, Henrica M. J. Werner, Jone Trovik, Anne M. Øyan, Karl-Henning Kalland, Inge Jonassen, Helga B. Salvesen, Kjell Petersen

**Affiliations:** 1 Computational Biology Unit, Department of Informatics, University of Bergen, Bergen, Norway; 2 Department of Obstetrics and Gynecology, Haukeland University Hospital, Bergen, Norway; 3 Bioinformatics and Systems Biology Program, School of Bioresources and Technology, King Mongkut’s University of Technology Thonburi, Bangkok, Thailand; 4 Centre for Cancer Biomarkers, Department of Clinical Science, University of Bergen, Bergen, Norway; 5 Centre for Cancer Biomarkers, Department of Clinical Medicine, University of Bergen, Bergen, Norway; 6 Department of Pathology, The Gade Institute, Haukeland University Hospital, Bergen, Norway; 7 Department of Microbiology, Haukeland University Hospital, Bergen, Norway; 8 Centre for Cancer Biomarkers, Department of Informatics, University of Bergen, Bergen, Norway; University of South Alabama Mitchell Cancer Institute, UNITED STATES

## Abstract

We have identified nine highly connected and differentially expressed gene subnetworks between aggressive primary tumors and metastatic lesions in endometrial carcinomas. We implemented a novel pipeline combining gene set and network approaches, which here allows integration of protein-protein interactions and gene expression data. The resulting subnetworks are significantly associated with disease progression across tumor stages from complex atypical hyperplasia, primary tumors to metastatic lesions. The nine subnetworks include genes related to metastasizing features such as epithelial-mesenchymal transition (EMT), hypoxia and cell proliferation. TCF4 and TWIST2 were found as central genes in the subnetwork related to EMT. Two of the identified subnetworks display statistically significant association to patient survival, which were further supported by an independent validation in the data from The Cancer Genome Atlas data collection. The first subnetwork contains genes related to cell proliferation and cell cycle, while the second contains genes involved in hypoxia such as HIF1A and EGLN3. Our findings provide a promising context to elucidate the biological mechanisms of metastasis, suggest potential prognostic markers and further identify therapeutic targets. The pipeline R source code is freely available, including permutation tests to assess statistical significance of the identified subnetworks.

## Introduction

Endometrial cancer is the most common female pelvic malignancy in industrialized countries. Even though three-quarters of the patients are treated at an early stage, approximately 20% of these recur after primary surgery [1]. In addition, women with metastatic disease have only 7–12 months median survival [2]. Improved patient treatment is within reach through better prediction of patients with high-risk for cancer recurrence and identification of therapeutic targets against metastatic diseases.

High-throughput technologies for studying global expression e.g. microarray or RNA sequencing are potential platforms allowing better understanding of the complexity of cancer biology. The study of global expression in metastatic endometrial cancer will lead to a better understanding of its processes and eventually identification of therapeutic targets. In the past decades, endometrial cancer studies were based only on tissue from primary lesions [3–5]. Here we perform a study of expression patterns within metastasizing tissue, with a focus on changes between aggressive primary tumors and metastatic lesions of endometrial cancer.

A wide range of computational approaches have been developed to extract biological meaning from gene expression data [6–10]. A typical analysis derives a list of significantly differentially expressed genes between two compared phenotypes. Subsequent biological interpretation involves investigating how these genes are related to biological processes and how they relate to each other. Two main types of approaches to analyze gene expression data are gene set enrichment analysis and network analyzes. Both have been proven successful in identifying distinct subsets of associated genes under study unraveling valuable biological information [11, 12]. As a natural consequence, many research groups have proposed different ways to integrate the two approaches to benefit from this complementarity [13, 14].

The gene set analysis facilitates biological interpretation by evaluating whether top ranked differentially expressed genes are enriched within pre-defined gene sets [11, 15, 16]. The gene sets are usually available in public databases and annotated for biological properties such as biological functions [17], pathway [18], and experimental models [19]. However, the gene set definitions will remain a critical factor for providing biologically meaningful results, and gene sets usually do not include any internal relations between the genes in each set [19].

The network analysis takes a different approach describing relations between biomolecules in the form of graphs or networks, where subnetworks can be derived as sets of nodes representing functional modules. Links in the network representing relations between biomolecules, are typically either based directly on experimental data assessing interactions (*e*.*g*. protein-protein interactions (PPI)) [20] or indirectly, for example through expression correlations [21]. These gene-gene or protein-protein relations provide more information inside the identified subnetworks aiding downstream interpretation. Several computational approaches have been developed to allow integration of different omics data through network analysis [22, 23]. In addition, the network topology itself can be derived from a particular condition and significant changes between the networks of two conditions could reflect molecular alterations and/or identify dysregulated modules [24, 25].

To investigate expression changes between the endometrial primary tumors and metastatic lesions, we set out to identify groups of related differentially expressed genes, complementing the typical differential expression analysis and facilitating biological interpretation by providing more information on gene-gene relationships. We have designed a new differential expression analysis pipeline that targets relatively small highly interconnected gene subnetworks. These are derived from a gene-gene correlation network captures the correlation between genes in either the primary tumor or metastasis sample group of our gene expression data set, and at the same time has been primed to emphasize *a priori* known gene-gene relations present in external resources collected by life science research community. This is achieved through a novel combination of the complementary network analysis and gene set analysis strategies. The network analysis serves the purpose of identifying subnetworks as well as integrating externally available gene-gene relations and internal (within the studied data) expression gene-gene relations. The gene set analysis evaluates the expression changes of the identified subnetworks between the studied conditions. Two established methods were modified and adapted into our proposed pipeline, Weighted Gene Co-expression Network Analysis (WGCNA) [21] and Gene Set Enrichment Analysis (GSEA) [11]. The use of phenotype specific gene subnetworks primed with *a priori* defined relations combined with enrichment analysis of differential expression, is as far as we are aware unique to our approach.

Our study presents a novel approach to identify expression changes that are biologically relevant for the metastasizing endometrial cancer. We have assessed the top ranked subnetwork results in light of current knowledge related to cancer biology and explored the gene expression profiles of the subnetworks in a larger set of patient samples for relations to clinical phenotypes e.g. disease progression stages and patient survival. The association between identified significant subnetworks and patient survival were also validated in another larger patient cohort from The Cancer Genome Atlas (TCGA) data [5].

## Materials and methods

### Materials

#### Patient series

The patients were diagnosed with endometrial carcinoma from 2001 to 2012 at Haukeland University Hospital, Bergen, Norway. Tumor tissue were prospectively collected and patients were staged according to FIGO 2009 criteria. Histologic subtype and grade, and follow-up data were obtained from clinical records [26]. All parts of the study have been approved according to Norwegian legislation. The study was approved by the Norwegian Data Inspectorate, Norwegian Social Sciences Data Services and the Regional Committee for Medical Research Ethics, REC West (NSD15501; REK 2009/2315 and REK 2014/1907). Participants gave written informed consent.

#### Gene expression data

RNA from 236 fresh frozen biopsies were extracted using the RNeasy Mini Kit (Qiagen, Hilden, Germany) and hybridized to Agilent Whole Human Genome Microarrays 44k (Cat.no. G4112F). Microarrays were scanned using the Agilent Microarray Scanner Bundle. The signal intensities were quantile normalized and log 2 transformed for each dataset separately, Dataset 1 for subnetworks identification and Dataset 2 for biological signal investigation of the detected subnetworks (See below). The raw and processed data have been submitted to ArrayExpress [E-MTAB-5017].

**For Dataset 1, a dataset for subnetwork identification**, we investigated gene expression patterns in 66 primary tumors from patients having metastasis at diagnosis or later recurrence, and 42 metastatic lesions. For 36 out of the 42 metastatic lesions, we had corresponding primary tumor samples from 26 individual patients. Of the 66 primary tumors, 24 were of non-endometrioid histology and 42 were of endometrioid subtype, of which 7, 14 and 20 were classified as histologic grade 1, 2 and 3, respectively (one case missing data for grade).

**Choice of samples to include in Dataset 1**; to assemble the most informative dataset with respect to the metastasis phenotype, we decided to utilize all samples available in our dataset, including some cases with multiple samples per patient at different progression stages. Limiting the samples to only matched pairs between primary tumors and metastatic lesions, would exclude the majority of the samples from the primary tumors and half of the samples from the metastasis group. The inter-sample relations introduced by including all samples, would introduce a systematic bias within sample group variance estimates in a traditional gene by gene differential expression analysis. As presented in Methods below, the gene by gene differential expression measure is in our pipeline simply used to rank the genes before a gene set analysis is performed on the top of this ranked list, so it is not likely that a similar systematic bias is introduced. The re-sampling to assess statistical confidence in our results is also not affected directly by this dataset sample composition, as it is done at the gene-gene level for both types of inputs to the pipeline.

**For Dataset 2, an expanded panel for biological signal investigation of the detected subnetworks**, 18 complex atypical hyperplasia (CAH) lesions and 110 primary endometrial carcinomas were additionally combined with Dataset1, and examined for associations between the identified subnetworks and clinico-pathologic features (S2 Table). In total, we investigated endometrial lesions from 18 hyperplasia with atypia, 176 primary carcinomas (34 non-endometrioid; 47, 52 and 40 endometrioid with grade 1, 2 and 3, respectively; three cases-data were missing for grade) and 42 metastatic lesions.

**Dataset 3, The Cancer Genome Atlas (TCGA) data**, was used to validate the association between detected subnetworks and disease specific survival as an independent patient validation cohort. We retrieved the RNA expression values from the Uterine Corpus Endometrial Carcinoma (UCEC), UNC IlluminaGA RNASeqV2 (Level 3) TCGA data. The expression data from 369 primary tumor samples were investigated for batch effects and mapped with clinical data to retrieve survival time annotation [5].

#### Protein-protein interaction (PPI) data

The protein-protein interaction (PPI) data were derived from CCBS Human Interactome Database [20] and used to infer *a priori* gene-gene relations. These are experimentally assessed physical interactions. The PPI data comprise 4625 proteins and 15958 interactions. 4361 proteins and 13329 interactions remained after mapping the proteins’ annotated gene names with the microarray data annotations.

### Methods

**Fig 1:**
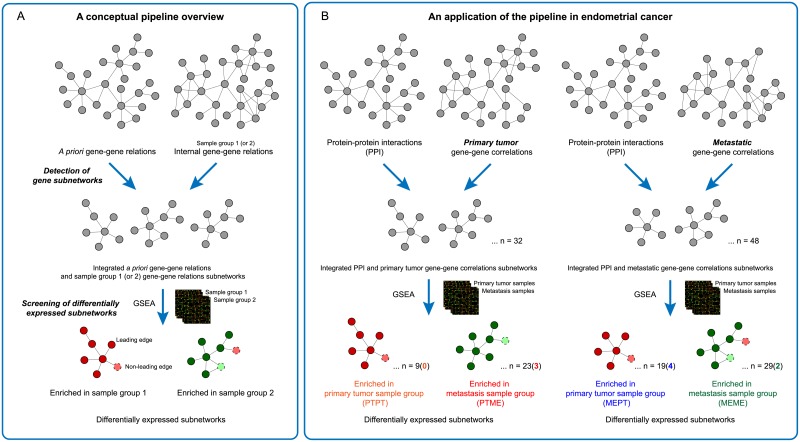
Analytical approach to identify differentially expressed subnetworks. A conceptual pipeline overview (A) and a specific application of the pipeline to our sample set of endometrial cancers (B) are shown, respectively. (A) First, the detection of gene subnetworks is performed by integrating *a priori* gene-gene relations and internal gene-gene correlations from one of the sample groups. Second, a screening of differentially expressed subnetworks between the two sample groups is carried out by applying GSEA to evaluate the detected candidate subnetworks for differential gene expression. Two ranked lists of differentially expressed subnetworks between the two studied sample groups are provided as results of the pipeline, containing subnetworks with differentially expressed genes found enriched in either sample group 1 or sample group 2. Permutation-based False Discovery rate (FDR) values are provided to reflect statistical confidence of the results, which controls for multiple testing of all identified subnetworks by the pipeline. The whole procedure is repeated again using gene-gene correlations of the second sample group as the basis for subnetwork detection in the first step. In total four lists of ranked differentially expressed subnetworks with FDR values are produced. (B) In our analysis, protein-protein interactions (PPI) are applied as *a priori* gene-gene relations. Internal gene-gene relations are computed as absolute Pearson correlations of expression between pairs of genes within the primary tumor and the metastasis sample group, separately. After evaluating these subnetworks for enrichment of differentially expressed genes in either primary tumors or metastatic biopsies, the four lists of subnetworks according to a starting gene correlation sample group combination with enrichment of up-regulated genes in a sample group, are represented at the bottom with different color labels: orange for subnetworks having primary tumor correlations between genes inside the subnetworks, and enriched in the primary tumor sample group (PTPT); red for subnetworks having primary tumor gene-gene correlations and enriched in the metastasis sample group (PTME); blue for metastatic gene-gene correlations and enriched in the primary tumor sample group (MEPT); and green for metastatic gene-gene correlations, enriched in the metastasis sample group (MEME).

We propose an analytical pipeline to identify highly connected and differentially expressed gene subnetworks (Fig 1A). The two main parts of the pipeline, (i) detection of gene subnetworks and (ii) screening of differentially expressed subnetworks, are described respectively. In addition, we explain statistical confidence estimates of the identified candidate subnetworks, permutation-based significance tests, as well as the analyses of the significant subnetworks with respect to clinical parameters.

#### Detection of gene subnetworks using *a priori* gene-gene relations and internal gene-gene relations

In order to identify subnetworks using both *a priori* gene-gene relations and internal gene-gene relations, we modified the WGCNA method, which is provided as an R package [21]. Following the protocol described in WGCNA [12], the first step is to define similarity between gene expression profiles across the studied samples. Let a similarity measure *s*_*ij*_ denote co-expression similarity of each pair of genes *i* and *j*. In this study, we used the absolute value of Pearson correlation

$$s_{ij}=|cor(i,j)|.$$

We used the soft threshold approach of WGCNA to transform *s*_*ij*_ by a power function with a power *β* ≥ 1 into an adjacency measure, scaling the connection between two genes. Here we measured the correlation within each sample group separately, primary tumors and metastatic lesions. In order to combine *a priori* gene-gene relations and internal correlations of the studied dataset, we re-defined an adjacency as a multiplication of the *a priori* gene-gene relations input and the transformed correlations

$$a_{ij}^{'}=p_{ij}\times s_{ij},$$

where *p*_*ij*_ represents an *a priori* relation of each pair of genes *i* and *j*. As we demonstrated the approach using PPI in this study, we defined *p*_*ij*_ = 1 if there is a PPI between gene *i* and gene *j*, otherwise 0. To derive $a_{ij}^{'}$, the parameter *β* was picked by optimizing the fit of the resulting transformed relations to the scale free topology criterion. This is a central point in WGCNA to ensure the naturally occurring connectivity pattern observed in biological networks, balancing the number of a few well connected central hub nodes versus many more rapidly decreasing connected nodes following a power law [27]. In this study, we picked power parameters *β* = 6 and *β* = 4 for soft thresholds of gene-gene correlation networks in the primary tumor and metastasis sample groups, respectively.

The adjacency matrix, a collection of our modified adjacencies between genes, was then used for subnetworks identification. First we computed the Topological Overlap Measure (TOM) [28] based on our modified adjacencies, and then we used TOM-based dissimilarity and hierarchical clustering with Dynamic Tree Cut [29] to divide the dendrogram into subnetworks (deepsplit = 2).

#### Screening of differentially expressed subnetworks

The derived subnetworks were then evaluated for their discriminatory power between two sample groups using GSEA. The expression data of both primary tumors and metastatic lesions were hence used in this step. The GSEAPreranked implementation by Broad Institute [11] enables the use of a pre-defined rank list of differentially expressed genes. We used Significance Analysis of Microarrays (SAM) [30] to pre-rank the genes according to differential expression between the aggressive primary tumors and metastatic lesions. In contrast to standard application of GSEA where gene sets from public data sources like Molecular Signatures Database (MSigDB) [19] or Gene Ontology (GO) [17] are applied, we used the identified subnetworks as the gene sets for the GSEA. Note that with the implementation of GSEAPreranked we could only perform gene-based permutation to estimate enrichment scores and confidence values. Nevertheless, there is a strong correlation between the normalized enrichment scores (NES) values generated from gene and sample-based permutation methods of GSEA (See supporting information S1 Fig). We limited the gene set size to be between 10 and 500 genes to gain an appropriate size for interpretation. After executing GSEA, ranked lists of enriched subnetworks in either primary tumors or metastatic lesions were shown with enrichment scores. The identified subnetworks were visualized using Cytoscape [31].

#### Permutation-based significance tests of candidate subnetworks

To evaluate the significance of the detected subnetworks, we performed permutation tests by re-executing the whole pipeline (as in Fig 1) using permutated input data. The two main input data of our subnetwork analysis are the *a priori* gene-gene relations and the internal gene-gene correlations. Each of these was permutated separately in two independent permutations tests. To permute the *a priori* gene-gene relations or PPI (referred to as PPI permutation), we kept the original PPI network topology constant, but randomly shuffled the gene labels between the nodes in the network. This gave new random PPI interaction partners, while the topology of the interaction networks was preserved. All other components than the input PPI, such as the gene expression data and workflow parameters, remained unchanged in this permutation test. For the permutation test of expression correlation input (referred to as gene permutation), we randomly re-assigned the gene labels in the expression data, keeping the underlying correlation structure of the data unmodified. This permuted expression data set, leading to a new set of internal gene-gene correlations, was used for both steps of subnetwork identification and the subsequent GSEA analysis. Analogously to the PPI permutation test, only the expression data input was permuted while all other factors such as PPI network and workflow parameter values were kept constant. We performed 500 permutations for each permutation test. The resulting NES from GSEA were used to compare the detected subnetworks to the permutation test generated subnetworks. Nominal p-values and False Discovery Rate (FDR) values were estimated from the comparison. The FDR values reflects statistical confidence of the results while taking into account the multiple testing of many subnetworks produced by the pipeline [32]. Note that these confidence measures pertain to results of the whole pipeline, and are not the same p-values or FDR values from the GSEA sub step of the pipeline.

#### Analysis of detected subnetworks with respect to clinical parameters

We investigated relationships between expression profiles of the significant subnetworks and clinico-pathologic parameters. For each subnetwork, we computed one signature score per sample, as the average of expression of leading edge genes after mean and variance normalization. We considered only leading edge genes as they by definition are the only genes contributing to the enrichment score [11].

The distribution of signature scores in relation to disease stages in the expanded sample series (Dataset 2) was visualized in boxplots and differences in the signature scores were measured across tumor groups by Mann-Whitney U test. The primary tumors were divided according to histologic type (endometrioid and non-endometrioid), and the endometrioid group was further divided according to histologic grade (1, 2 and 3).

Kaplan-Meier survival analysis was applied to compare disease specific survival (the number of deaths resulting from endometrial carcinoma) between patient groups (*e*.*g*. cases with high and low subnetwork signature scores). Cut-offs for signature scores were determined by using quartile values (Q1-4) and the groups with similar survival were merged (*e*.*g*. Q1-2 vs Q3-4 or Q1-3 vs Q4). Survival differences between groups were estimated using the log-rank test. The survival differences of subnetworks passing Bonferroni multiple testing corrections with significant p-values (p < 0.05) are shown as results.

## Results

### Our pipeline identifies highly connected and differentially expressed subnetworks

We have developed a novel pipeline to identify highly connected and differentially expressed gene subnetworks. The analytic pipeline was implemented in R and deposited in the GitHub repository, https://github.com/diffsubnet/codes. A conceptual pipeline overview is shown in Fig 1A, and an application of the pipeline to analyze endometrial cancer gene expression data is shown in Fig 1B. The pipeline enables high-throughput data analysis and interpretation by identifying candidate subgroups of functionally related genes and displaying their gene-gene relations as a subnetwork. The method was designed to combine publicly available external gene-gene relations with internal gene-gene expression correlations of the data set under study, to target differential expression changes in gene modules specific to the conditions of interest. WGCNA [12] was modified to integrate the two sources of gene-gene relations for deriving subnetworks, and then GSEA [11] was applied to evaluate differential expression between two sample groups utilizing the identified subnetworks as gene sets.

In this study, we used PPI as external or *a priori* gene-gene relations and employed the pipeline to study genes differentially expressed between 66 aggressive primary tumors and 42 metastases in endometrial cancer. By integrating information from PPI databases both with primary tumor gene-gene correlations and metastatic gene-gene correlations, we detected 32 and 48 highly connected subnetworks, respectively (Fig 1B). The integrated subnetworks show scale free topology property as the original method with good fits of R^2^ ≥ 0.9 (see supporting information S1 Text). By evaluating differential expression of the identified subnetworks, the subnetworks were categorized according to their enrichment of up-regulated genes in either of the two sample groups, primary tumors or metastatic lesions. Four lists of subnetworks were derived according to their initial gene-gene correlations source and differential expression enrichment: subnetworks having primary tumor gene-gene correlations and enriched in primary tumor sample group (PTPT); subnetworks having primary tumor gene-gene correlations and enriched in metastasis sample group (PTME); subnetworks having metastatic gene-gene correlations and enriched in primary tumor sample group (MEPT); or subnetworks having metastatic gene-gene correlations and enriched in metastasis sample group (MEME). The detected subnetworks in each category were provided with their enrichment scores (ES) and NES from GSEA. The top five ranked subnetworks in each category are shown in Table 1.

**Table 1:** The differentially expressed subnetworks between primary tumors and metastatic lesions in endometrial cancer with permutation scores. The top five ranked subnetworks of each subnetwork category according to gene-gene expression correlation types and enrichment groups are shown with their sizes (numbers of genes in the subnetworks), enrichment scores (ES) and normalized enrichment scores (NES) from GSEA, and permutation scores. PPI and gene permutations indicate the permutation of *a priori* gene-gene relations and internal gene-gene correlations, which are the two main inputs for defining subnetworks, respectively. Nominal p-value and False Discovery Rate (FDR) are provided as significance measures.

Subnetwork name	Size	ES	NES	PPI permutation		Gene permutation	
				p-value	FDR	p-value	FDR
Primary tumor gene-gene correlations, enriched in primary tumor sample group (PTPT)							
PTPT1	20	-0.44	-1.39	0.1366	1.0000	0.1234	1.0000
PTPT2	11	-0.51	-1.35	0.1601	0.7205	0.1443	0.6494
PTPT3	16	-0.42	-1.24	0.2292	0.6875	0.2142	0.6426
PTPT4	30	-0.31	-1.09	0.3799	0.8548	0.3608	0.8118
PTPT5	17	-0.34	-1.04	0.4381	0.7886	0.4191	0.7544
Primary tumor gene-gene correlations, enriched in metastasis sample group (PTME)							
PTME1	11	0.80	2.06	0.0030	0.0693	0.0027	0.0622
PTME2	65	0.47	1.85	0.0194	0.2230	0.0188	0.2157
PTME3	20	0.58	1.77	0.0378	0.2898	0.0350	0.2683
PTME4	25	0.49	1.57	0.1163	0.6689	0.1161	0.6675
PTME5	20	0.51	1.54	0.1380	0.6348	0.1365	0.6277
Metastatic gene-gene correlations, enriched in primary tumor sample group (MEPT)							
MEPT1	20	-0.61	-1.96	0.0011	0.0206	0.0011	0.0211
MEPT2	17	-0.60	-1.82	0.0041	0.0393	0.0044	0.0413
MEPT3	16	-0.56	-1.69	0.0136	0.0861	0.0136	0.0833
MEPT4	25	-0.48	-1.65	0.0167	0.0795	0.0181	0.0860
MEPT5	42	-0.33	-1.31	0.1349	0.5128	0.1390	0.5282
Metastatic gene-gene correlations, enriched in metastasis sample group (MEME)							
MEME1	30	0.57	1.90	0.0031	0.0911	0.0039	0.1134
MEME2	14	0.65	1.83	0.0070	0.1018	0.0071	0.1035
MEME3	39	0.46	1.63	0.0348	0.3360	0.0313	0.3025
MEME4	18	0.54	1.58	0.0473	0.3426	0.0423	0.3064
MEME5	14	0.57	1.56	0.0563	0.3264	0.0510	0.2957

### Nine highly connected and differentially expressed subnetworks between primary tumors and metastatic lesions in endometrial cancer were detected and statistically significant

We performed permutation-based significance tests to evaluate the statistical significance of the detected subnetworks. We compared the NES values of the detected subnetworks with the NES values of subnetworks generated by the pipeline with one permuted input source at a time, PPI data and gene-gene expression correlations (PPI and gene permutations), respectively. Table 1 shows the top five ranked subnetworks with permutation-based p-values and FDR values for each of the four lists of subnetworks (PTPT, PTME, MEPT and MEME). For both PPI and gene permutation results, the originally observed subnetworks had significantly higher NES than the subnetworks generated from permutated data.

**Fig 2:**
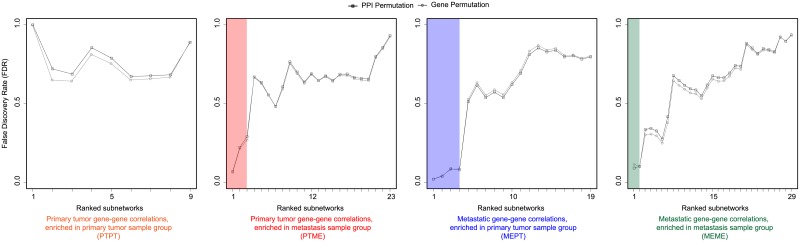
Nine statistically significant subnetworks detected in endometrial cancer. For each of the four lists of identified subnetworks in Fig 1B, we have plotted the ranked subnetworks by decreasing score versus their corresponding FDR values. The two permutation-based tests for estimating FDR values are performed by evaluating the observed scores of the ranked subnetworks in contrast to the scores obtained when the main input data of PPI (PPI permutation) and gene-gene expression correlation (Gene permutation) are randomly permuted, respectively. We selected the top ranked subnetworks before the first substantial increase in FDR values, as significant findings for further investigation. The significant subnetworks are marked with colored boxes: PTME (n = 3, FDR_PPI_ = 0.29, FDR_Gene_ = 0.27) in red; MEPT (n = 4, FDR_PPI_ = 0.08, FDR_Gene_ = 0.09) in blue; and MEME (n = 2, FDR_PPI_ = 0.10, FDR_Gene_ = 0.10) in green. PTPT did not have any significant subnetworks.

**Fig 3:**
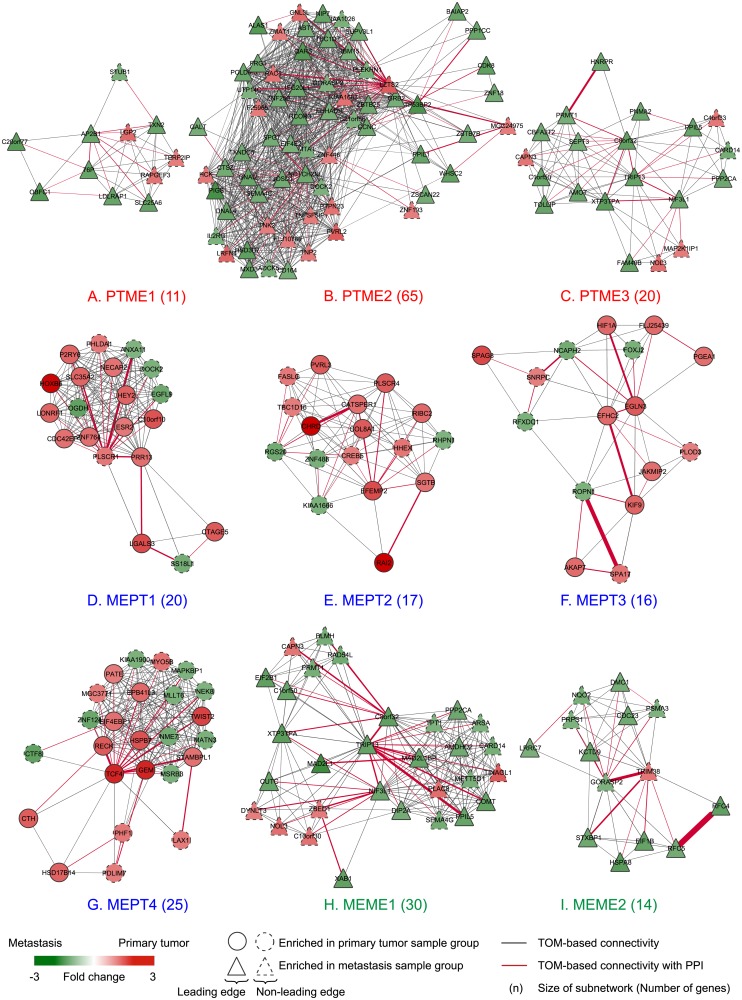
Nine significant differentially expressed subnetworks between primary tumors and metastatic lesions in endometrial cancer. Subnetworks identified from PPI and gene-gene expression correlations in primary tumors; PTME (A-C), and metastases; MEPT and MEME (D-I) are shown, respectively. Subnetworks marked as circular nodes are enriched with up-regulated genes in primary endometrial carcinomas while triangular nodes indicate enrichment of up-regulated genes in metastatic lesions. In each subnetwork, a node represents a gene and its corresponding protein, and a link between two nodes represents Topological Overlap Measure (TOM)-based connectivity. The thickness of a link shows levels of TOM-based connectivity and a red colored link represents two nodes with a PPI between them. For each node, the colors show fold change of expression between primary tumors and metastatic lesions. Red color indicates higher gene expression in primary endometrial carcinomas and green color indicates higher gene expression in metastatic lesions. Leading edge nodes are illustrated with solid lines, while non-leading edge nodes have dashed lines. The nine subnetworks are shown with colored labels according to a starting sample group of gene-gene correlations and enrichment of up-regulated genes in a sample group: PTME in red; MEPT in blue; and MEME in green.

**Fig 4:**
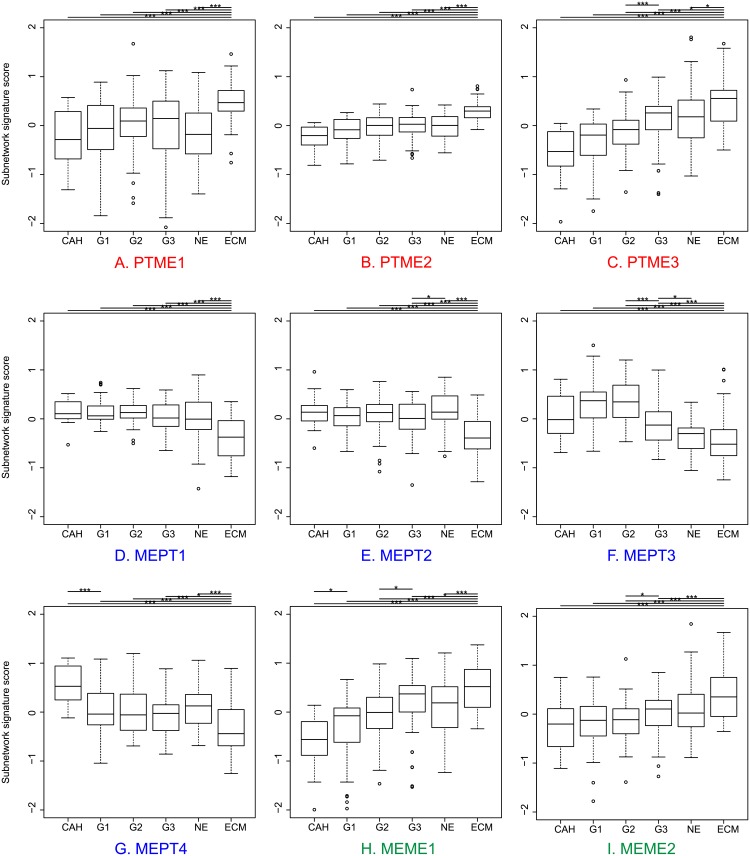
The significant subnetworks display a steady trend of gene expression signature scores according to disease progression stages. Boxplots show signature scores for the nine detected subnetworks relative to stages of disease progression. The stages are from left to right are: complex atypical hyperplasia (CAH), endometrioid primary tumors from grade 1 through 3 (G1, G2 and G3), non-endometrioid primary tumors (NE), and metastatic lesions (ECM). Annotations above boxplots indicate levels of significant difference between signature scores in different disease stages; p-values < 0.05* and 0.005***. Color labels indicate subnetworks categories (see description in Fig 1): red color: PTME (A-C); blue color: MEPT (D-G); green color: MEME (H-I).

To suggest top ranked subnetworks for interpretation and further investigation, we investigated the FDR values from the permutation test results. We found that at particular points in the ranked subnetwork lists of decreasing scores (NES), the FDR values distinctly increased for both permutation tests. We used this criterion to prioritize among the detected subnetworks for further investigation. Fig 2 displays FDR values for each ranked resulting subnetwork. Note that the identified subnetworks were ranked according to their NES from GSEA. For example, from rank four to five in the MEPT subnetworks, the FDR values substantially increase (from 0.0795 to 0.5128 and 0.0860 to 0.5282 for PPI and gene permutations, respectively), resulting in four significant subnetworks from the MEPT result list. So, we considered the top three, four, and two of subnetworks in PTME, MEPT and MEME result lists, respectively for further analysis. We did not investigate PTPT subnetworks further, due to very high FDR values (FDR = 1). The nine selected subnetworks, using the criterion of substantially increased FDR value as a natural cut-off limit, are displayed in Fig 3. We display each category of subnetworks with different color labels, which are orange (PTPT), red (PTME), blue (MEPT) and green (MEME). The same color coding is used through Figs 1 to 4.

In addition, our proposed pipeline can detect genes that are not significantly differentially expressed at an individual level, but are significantly differentially expressed as a group. In fact, the majority of the genes in the nine detected subnetworks do not show individually significant differences between the two sample groups (S1 Table).

### Subnetwork gene expression patterns correlate with disease progression and disease specific survival

**Fig 5:**
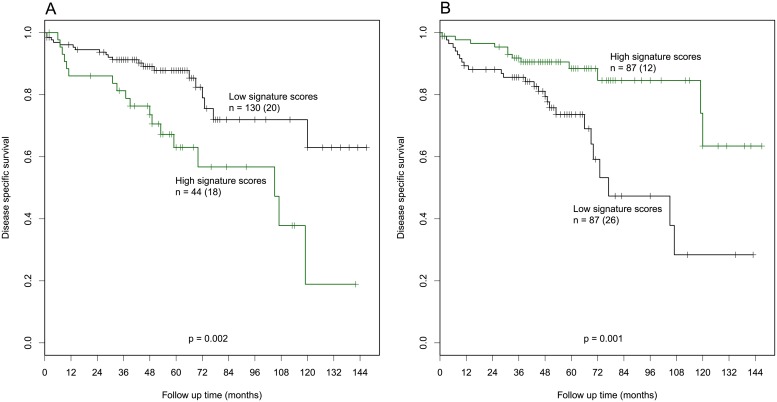
Two out of nine detected subnetworks are significantly associated with disease specific survival. Kaplan Meier survival analysis plots display disease specific survival according to gene signature scores of the (A) PTME3 (Quantile values of Q1-3 vs Q4 for the patient groups of low and high signature scores, respectively; p = 0.002) and (B) MEPT3 (Q1-2 vs Q3-4 for the groups of low and high signature scores, respectively; p = 0.001) subnetworks. The groups of high signature score in PTME3 subnetwork and low signature score in MEPT3 subnetwork associate with poorer outcomes. The numbers of patient (n) in each group are displayed with the number of deaths resulting from endometrial carcinoma in parenthesis. Both subnetworks have features previously reported to be essential for aggressive tumor biology: In the PTME3 subnetwork the majority of genes are linked to regulation of cell cycle and proliferation. In the MEPT3 subnetwork, central nodes are related to hypoxic response.

We investigated gene expression profiles of the nine significant subnetworks (Fig 3) in the expanded sample series (Dataset 2; see Materials), including CAH and additional primary tumor samples (S2 Table). The detected subnetworks show patterns of signature scores in Dataset2 consistent with the expression changes in the subset of samples from which the subnetworks were primarily identified. The patterns display trends of continuously increasing or decreasing signature scores relative to disease progression (Fig 4). The stages of progression is defined from less to more aggressive as CAH, endometrioid primary tumors from grade 1 through 3, non-endometrioid primary tumors, and metastatic lesions, respectively. Furthermore, two out of nine subnetworks, PTME3 and MEPT3 (Fig 3C and 3F), are significantly associated with disease specific survival when comparing patient groups with high and low signature scores within each subnetwork (Fig 5A and 5B, respectively). We report in more details on each of the nine submodules according to their gene-gene correlations and expression enrichment below.

The PTME subnetworks (Fig 3A–3C) show trends of increasing signature scores within endometrioid tumors with increasing histologic grade and also higher scores in non-endometrioid tumors compared to endometrioid tumors, indicating higher score the more aggressive disease (Fig 4A–4C). This is consistent with regard to gene expression of the detected subnetworks that are up-regulated in the metastatic compared to the primary lesions. All three subnetworks have significantly higher signature scores in metastases compared to other disease stages. In addition, PTME3 displays significant association with disease specific survival between patient groups with low and high signature scores (Quantile Q1-3 vs Q4, respectively; p = 0.002). The patients with high signature scores have relation to poorer outcome.

For the MEPT subnetworks (Fig 3D–3G), the signature scores of the metastasis sample group are significantly lower compared to the other groups (Fig 4D–4G). The MEPT1 and MEPT2 subnetworks show the same pattern, with strongly decreasing signature scores in the metastases (p<0.005). However, only slight differences can be seen among the other groups (Fig 4D and 4E). The MEPT3 and MEPT4 reflect more unique expression patterns. The MEPT3 submodule (Fig 3F) displays significantly lower levels of signature scores between primary tumors from grade 2 (G2) to grade 3 (G3) and non-endometrioid (NE) group (Fig 4F, p<0.05). Furthermore, the subnetwork shows association with disease specific survival when compare between patient groups with high (Quantile Q1-2) and low (Q3-4) signature scores. The low score group associates with poorer outcomes. For the MEPT4 subnetwork (Fig 3G), the boxplot (Fig 4G) elucidates significantly higher signature scores in precursor lesions and dropping in primary tumors and metastases.

The MEME1 and MEME2 subnetworks (Fig 3H and 3I) show significantly increasing signature scores from hyperplasia to primary tumors and metastases (Fig 4H and 4I), extrapolating the trend from the expression differences used to rank the subnetworks. The signature scores in both subnetworks show similar patterns with increasing values from hyperplasia, primary tumors to metastases, indicating that the majority of genes in these submodules are up-regulated in aggressive disease. In addition, we found that several genes in the MEME1 subnetwork overlaps with the PTME3 subnetwork (TRIP3, C8orf32, NIF3L1, XTP3TPA, C1orf50, PRMT1, CAPN3, PPP2CA and CARD14) showing that these genes have strong correlations both in metastatic lesions and primary tumors, and are up-regulated in metastases.

### The association between expression patterns of the identified subnetworks and disease specific survival is validated in TCGA cohort

The significant correlation between the identified PTME3 and MEPT3 subnetworks and disease specific survival was validated in the TCGA data set of 369 endometrial carcinoma cases. The signature scores of the PTME3 subnetworks in the TCGA data set (low vs high signature score groups: quantile Q1-3 vs Q4) display a solid significant association with disease specific survival (p<0.000001). The high signature scores associate with poorer outcomes compared to the low signature scores, corresponding to the results shown in our cohorts. The MEPT3 subnetwork shows a non-significant trend (p = 0.12) toward disease specific survival association (quantile Q1 vs Q2-4 for the patient groups of low and high signature scores, respectively), however the trend matches the results in our studied cohorts that the low signature scores (Q1 quantile) identify patients with poorer survival. Together, these results of the external TCGA cohort supports a potential biological relevance of the identified subnetworks (S4 Fig).

## Discussion

### The identified subnetworks relate to epithelial-mesenchymal transition (EMT), hypoxia and cell proliferation

In all nine detected subnetworks, we found several genes that previously have been reported as relating to carcinogenic processes such as epithelial-mesenchymal transition (EMT), hypoxia and cell proliferation. These are processes important for tumor growth and progression. Even though the annotations of many genes are incomplete or rather generic, the focused set of genes and their relations in the subnetworks may lead to more clues for interpretation and further studies in endometrial cancer especially in the metastatic setting. We discuss some interesting biological findings and hypotheses based on the detected subnetworks below.

The signature scores of the PTME subnetworks increase with disease stage and increasing tumor aggressiveness. Up-regulation of genes in these submodules could suggest an important role in cancer progression. Leading edge genes in these subnetworks could be considered for further investigation as novel treatment targets for patients with endometrial metastatic lesions since they are together up-regulated in metastases compared to primary tumors. Many genes are reported as related to cell cycle and cell proliferation. In the PTME3 subnetwork (Fig 3C), the majority of genes are related to cell proliferation and cell cycle processes, for instance, TRIP13, PPP2CA, SEPT3, PRMT1, HNRPR and C8orf32 [33, 34]. Sustained proliferation is suggested as necessary for tumor growth, and as such a hallmark of cancer [35]. More aggressive tumors have a stronger proliferative signal. High proliferation, as assessed by other biomarkers (*e*.*g*. Ki67 immunostaining), has previously been associated with tumor aggressiveness and reduced survival [36] as also supported by our data (see Results). Enrichment of subnetworks reflecting proliferation in the metastatic lesions is novel knowledge in the field of endometrial cancer research. The micro-metastatic lesion needs proliferative activity to grow into a macro-metastasis [37], and our results support such activity in the metastatic endometrial lesions. As the signature score of the PTME3 subnetworks show significant association to disease specific survival, the potential of this subnetwork as a prognostic marker in endometrial carcinoma should be further explored.

The signature scores of the MEPT subnetworks are decreasing significantly from precursor lesions and primary tumor to metastatic lesions, suggesting genes in these networks to be important for the invasive properties of the aggressive primary tumors. In clinical perspective, these gene groups could be further studied for detecting the signal of aggressive endometrial tumors. Interestingly, in the MEPT3 submodule, central nodes like HIF1A and EGLN3 are related to hypoxia [38–40]. HIF1A is a major transcription factor that activates a broad range of genes important for the cellular response to hypoxia, which has downstream effects including angiogenesis and glucose metabolism [41]. Hypoxic conditions are known to promote tumor development and invasion, features essential in an aggressive primary tumor and tumor progression [42]. Central and leading edge nodes in the MEPT4 subnetwork (Fig 3G), such as TCF4 and TWIST2, are well known players of EMT [43, 44]. The boxplot in Fig 4G reveals that the leading edge genes are highly expressed in both the precursor lesions and the highly aggressive non-endometrial tumors, but lower expressed in metastases. The changing pattern of expression toward aggressive disease could be explained by changing stages of EMT during different stages of the carcinogenic process [45]. Newly colonized metastatic cells are often in a proliferative stage where they undergo the opposite transition, mesenchymal–epithelial transition (MET) [46], in concordance with our observation of lower expression of EMT-related genes in the metastatic lesions.

The signature scores of the MEME subnetworks increase with disease progression and aggressiveness. Several genes in these subnetworks are related to cell cycle and cell proliferation process [47, 48]. Genes in the MEME1 subnetwork, such as MAD2L1, PRMT1 and GPN1 have previously been reported to be up-regulated in metastases compared to primary tumors [49], consistent with our finding. The genes up-regulated in the MEME2 subnetwork (Fig 3I), RFC4, RFC5 and CDC23 has been reported associate with lymph node metastases in endometrioid endometrial tumors [50].

### Statistical significance of the identified subnetworks

We performed a non-parametric statistical test to assess the significance of the identified subnetworks from the whole workflow. As the introduced concept of integrating *a priori* gene-gene relations and internal gene-gene correlations for detecting subnetworks is the essence of this approach, we permuted the two main sources of input data, PPI and gene expression. When designing both permutation tests, we made sure to keep essential features of the original data to generate a realistic background distribution of permuted data. In both tests the network topology was kept unchanged, while all original gene-gene relations were broken and replaced by random new partners. The results in Table 1 show that disrupting the gene-gene relations only in one input data source alone is sufficient to generate permuted data that generally evaluates worse than the original data. This emphasizes that both input data sources are important for the detection of the significantly differentially expressed subnetworks.

Furthermore, the derived FDR values from PPI and gene permutation tests were only slightly different. Since coherence between gene-gene relations in both PPI and correlation data for a given gene pair is necessary to contribute to a successfully evaluated subnetwork, randomizing either data type for a given pair could be assumed to lead to similar results. The permutation test results show that both data sources contribute substantially to the pipeline for detecting differentially expressed subnetworks. Finally, note that by keeping the network topology of the input data unchanged, the permuted data is expected to evaluate with higher enrichment scores than more randomized data with altered network topology. Thus, more favorable p-values and FDRs could be generated from more freely randomized permutation data, but this would in our opinion represent a less realistic background distribution to compare against.

### The features of our proposed pipeline

#### A combination of network and gene set analysis approaches

Available gene set and network approaches have been combined in several studies. Kong *et al*. [51] show an example of using network analysis results and gene set analysis results for interpretation to support each other, while many studies performed network analysis and used gene set analysis on top of the identified subnetworks for functional interpretation. For instance, Lui *et al*. and Fang *et al*. [52, 53] identified well connected subnetworks in the gene interaction network, and then applied gene set analysis to find enrichment of functional terms in the detected subnetworks using a set of publicly available functionally annotated gene sets, adding external validity to the biological relevance of their finding. In addition, several tools and frameworks have been developed to facilitate such use of both approaches sequentially in a pipeline [13, 14]. Our proposed pipeline combines the features of network analysis and gene set enrichment approaches in a more closely coupled manner towards a different purpose. The network analysis serves the purpose of identification of subnetworks and integration of externally available gene-gene relations and internal expression gene-gene relations, and the gene set analysis evaluates expression changes of the subnetworks between the studied conditions. To our knowledge, mRNA expression data have never before been combined in such a manner to reveal tumor phenotypic differences.

In a pure gene set-based method, the resulting ranked gene sets have by definition no internal relations between the genes [11], and the gene sets are the exact same sets provided as input to the algorithm, i.e. they are not adapted to any expression connectivity patterns present in the data. In our method, the produced gene subnetworks display two types of relations (TOM and PPI connectivity), and the set of genes evaluated as a group by GSEA is derived from the connectivity pattern of the expression data, where well connected hubs will act as seeds of subnetworks.

A pure network-based method that derives a network only from expression correlations, such as WGCNA, yields many more relations between genes without any external weight of importance [12]. In our development work, generating subnetworks using gene correlations only as input produced many larger subnetworks. In the presented results, we obtained smaller subnetworks integrating both experimentally verified physical interactions and internal gene-gene expression correlations that together helped narrow down gene-gene relations for further interpretation.

Furthermore, even if we used PPI as *a priori* gene-gene relations in this study, we suggest that other kinds of *a priori* gene-gene relations can be explored using the same pipeline concept. Also, the approach itself is not limited to RNA expression data, and could be applicable to other types of omics data. However, this would require further investigation on appropriate gene-gene relation metrics as inputs to the WGCNA network analysis methods.

#### The modified WGCNA and integration of gene-gene relations sources

The core feature of the suggested pipeline is to let *a priori* gene-gene relations guide the analysis to take into account existing evidence of possible functional relations between genes, while the actual relations of the genes under the given expression conditions are derived from the gene-gene correlations. Similar combinations of multiple levels of gene-gene relations are commonly explored for network constructions [54] and co-clustering approaches [22]. The work of Ulitsky and Shamir [22] integrated PPI and gene correlations in a manner similar to our approach before further downstream analysis to identify subnetworks. We found the WGCNA method suitable for integration of the two sources of gene-gene relations, as this allows the correlation network topology to define relation strengths using TOM, at the same time facilitating a clean integration with the PPI network information to emphasize the importance of the gene-gene relations annotated *a priori* as possibly functionally related.

The scale free topology criterion is an important prerequisite for WGCNA to be able to partition a network into highly connected subnetworks based on connectivity topology. When combining two sources of gene-gene relations, the overlapping genes will get an enhanced focus. In this work, the PPI data has significantly fewer genes and interactions compared to the correlation data, partly because the PPI data is expected to still be incomplete [20]. We note that we could accomplish a better interpretation of biological findings once we have more PPI annotations. However, the method itself directly supports more complete relation data. Too few relations would also make it hard for a network to achieve a good fit to the scale free topology criterion, but we achieved satisfactory fits (R^2^ ≥ 0.9) for the combined network of our method applied to the data set under study.

#### The use of GSEA

GSEA is a versatile tool in several aspects. It is very flexible in how gene sets are defined and hence supports many sources of gene sets [11, 19]. A possible drawback of this flexibility is the use of too many public sources of gene sets of varying quality and curation, leading to challenges regarding multiple testing and little structured documentation to follow up on selected gene sets. In this work, we use the correlation structure of the data itself (only one sample group at the time) to emphasize relations supported by pre-existing gene-gene relation data, and use the weighted connectivity network to define a relatively limited set of highly connected subnetworks specific to the underlying data as gene sets. Hence, we use GSEA in the alternative way suggested in the original Subramanian *et al*. paper [11] purely as a mean to screen user-defined gene sets for differential expression, to see if a predicted subnetwork as a set of genes appears to be differentially expressed compared to the global list of genes, not for functional annotation evaluation. The results in Table 1 show that this targeted approach yields low FDR values for the top networks in 3 out of 4 possible result categories (combinations of correlation sources and enriched sample group).

In addition, GSEA does not utilize a differential expression score cut-off when evaluating each gene set for differential expression. Instead, it evaluates the rank distribution of all member genes in a gene set in the ranked background list of all genes according to a differential expression score. Consequently, all genes in a resulting subnetwork may be included and displayed in their context, and as the genes are evaluated together as a set they do not necessarily have a strong individual score. In our detected subnetworks, many genes that are not individually differentially expressed demonstrate relevant biological processes and lead to more clues for interpretation of underlying mechanisms.

## Conclusions

In conclusion, we present a new analysis approach for differential network biology, allowing combination of external gene-gene relations with internal co-expression data, enabling detection of condition-specific and differentially expressed subnetworks. The method includes a conservative permutation-based method for assessing statistical significance of the resulting subnetworks. By applying the proposed algorithm on gene expression data of primary tumors and metastatic lesions in endometrial cancer, we detected nine subnetworks with significant enrichment scores. The contained genes display several interesting leads with respect to metastatic endometrial carcinoma. First, the expression differences identified by our pipeline show consistent patterns relative to disease progression when assessed across a larger panel of precursor lesions, primary tumors and metastatic lesions. Secondly, two of the detected subnetworks demonstrate strong associations to disease specific survival, also supported by validation results in the independent TCGA cohort. Third, several expected hallmarks of cancer were found, in manners consistent with disease stages. Thus, our method has successfully identified biologically relevant results in the endometrial cancer data set under study, which is also supported by independent data, and a mean for prioritizing central and driving genes of these subnetworks as candidates for further follow up. Taken together, this indicates that the proposed network analysis approach can be useful to detect highly connected and differentially expressed subnetworks of particular relevance for the phenotypes under study.

## Supporting information

S1 Text Scale free topology property of the integrated subnetworks. (DOC)

S1 Fig Correlation of normalized enrichment scores (NES) between gene and sample-based permutation methods of GSEA. Plots show correlation of NES between gene and sample-based permutation of GSEA of the identified subnetworks. The correlation plots of four types of subnetworks derived according to their initial gene-gene correlations source and enrichment of up-regulated genes in a sample group are shown respectively: (A) subnetworks having primary tumors gene-gene correlations and enriched in primary tumor sample group (PTPT); (B) subnetworks having primary tumors gene-gene correlations and enriched in metastasis sample group (PTME); (C) subnetworks having metastatic gene-gene correlations and enriched in primary tumor sample group (MEPT); (D) subnetworks having metastatic gene-gene correlations and enriched in metastasis sample group (MEME).
(PDF)

S2 Fig Scale free topology plot. Plots between power parameters of soft threshold vs scale free topology fit and mean connectivity, respectively. The plots of primary tumor (A) and metastasis (B) correlations integrated with PPI are shown, respectively. For each correlation type, plots between power parameters and scale free topology fit (left), and plots between power parameters and mean connectivity (right) are shown. The top panel shows results from different number of bins (from 3 to 10) using for scale free topology fit (1 represent 10 in this plot), while the bottom panel shows results with the optimal number of bin (the biggest bin number while the fit R^2^ ≥ 0.8). The numbers in the plots represent the numbers of bins except the bottom right plot that the numbers represent power parameters.
(PDF)

S3 Fig TOM plots. TOM plots of primary tumor (A) and metastatic (B) correlations, respectively. The plots are after integration of the expression correlations and PPI.
(PDF)

S4 Fig Disease specific survival in TCGA cohort. Disease specific survival analyzes according to gene signature scores of the (A) PTME3 (Quantile values of Q1-3 vs Q4 for the patient groups of low and high signature scores, respectively) and (B) MEPT3 (Q1 vs Q2-4 for the groups of low and high signature scores, respectively) subnetworks in TCGA endometrial data. The numbers of patient (n) in each group are displayed with the number of deaths resulting from endometrial carcinoma in parenthesis.
(PDF)

S1 Table A ranked list of differentially expressed genes. Table shows a ranked gene list from ordinary differentially expressed genes (SAM, FDR < 0.01) and the genes overlapped with genes in our detected subnetworks.
(XLS)

S2 Table Dataset for subnetworks identification (Dataset 1) and for biological signal investigation (Dataset 2). The table shows a number of samples used in the Dataset 1, a dataset for subnetwork identification, and Dataset 2, an expanded panel for biological signal investigation of the detected subnetworks. The progression stages from low to high disease aggressiveness are displayed from left to right; complex atypical hyperplasia (CAH), endometrioid primary tumors (ECPT) from grade 1 through 3 (G1, G2 and G3), non-endometrioid primary tumors (NE), and metastatic lesions (ECM), respectively.
(PDF)
