# Supplementary material for: Identification of highly connected and differentially expressed gene subnetworks in metastasizing endometrial cancer
Source: PLoS One. 2018 Nov 1;13(11):e0206665. doi: 10.1371/journal.pone.0206665 (PMC6211718; doi:10.1371/journal.pone.0206665)
Supplement: S1 Text — (DOC) [file pone.0206665.s001.doc]

**S1 Text. Scale free topology property of the integrated subnetworks.**

The modified WGCNA allows integration of two sources of gene-gene relations while maintaining scale free topology property. The connectivity between genes was redefined from the original WGCNA method for detection of highly connected subnetworks by combining *a priori* gene-gene relations with internal gene-gene relations as a weighted product. With PPI as the *a priori* gene-gene relations source, and a strict boolean overlap criterion for combining with the internal relations, the number of links between genes is mainly limited by the PPI data. Despite the reduction in the number of links, the combined network still produced good fits (R² ≥ 0.9) towards the scale free topology criterion central in WGCNA (S2 Fig). Nevertheless, we found that the fit R² value heavily depended upon the exact number of bins for dividing the adjacency measures, most likely because of a more uneven distribution of adjacency values compared to regular correlation networks normally used with WGCNA. We kept the number of bins as close to the default while avoiding unintended side effects of the adjacency binning procedure. A more even distribution of adjacency values could be sought after through alternative formulas for integrating *a priori* gene-gene relation information with the internal correlation data. The TOM-based dissimilarity was calculated and hierarchical clustering with Dynamic Tree cut was applied to identify subnetworks. The TOM plots are provided in S3 Fig.
