# Supplementary figures and images for: Identification of highly connected and differentially expressed gene subnetworks in metastasizing endometrial cancer
Source: PLoS One. 2018 Nov 1;13(11):e0206665. doi: 10.1371/journal.pone.0206665 (PMC6211718; doi:10.1371/journal.pone.0206665)

A

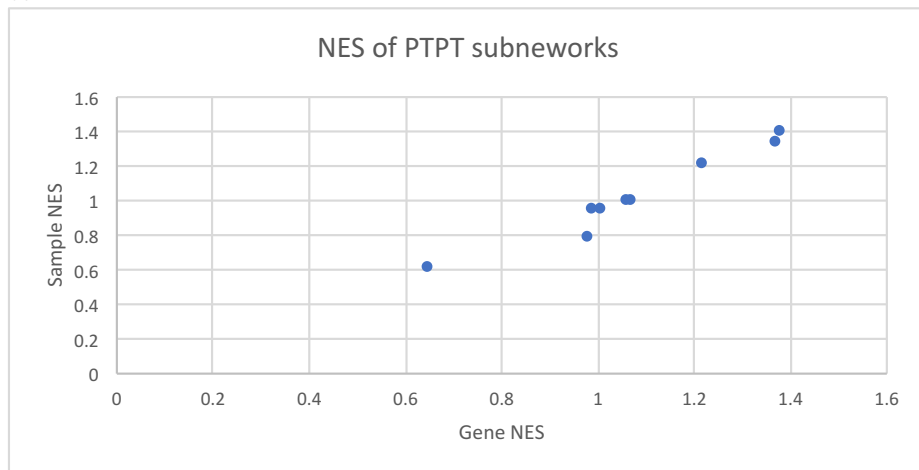

B

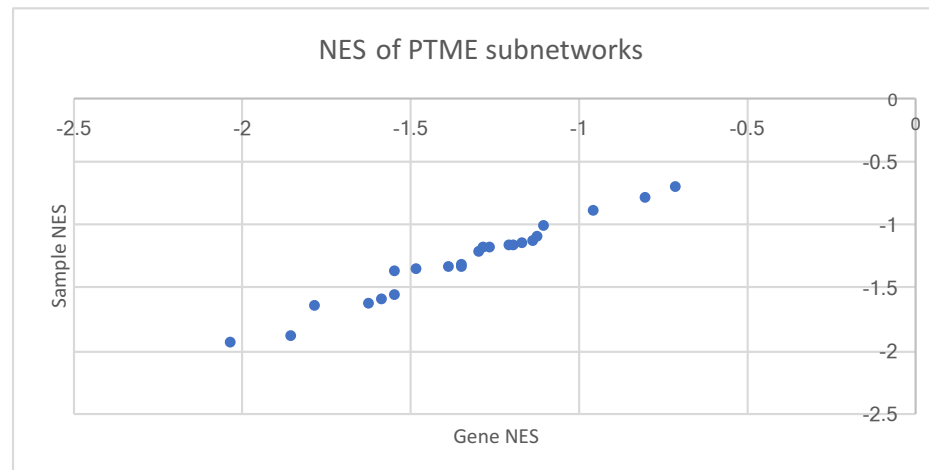

C

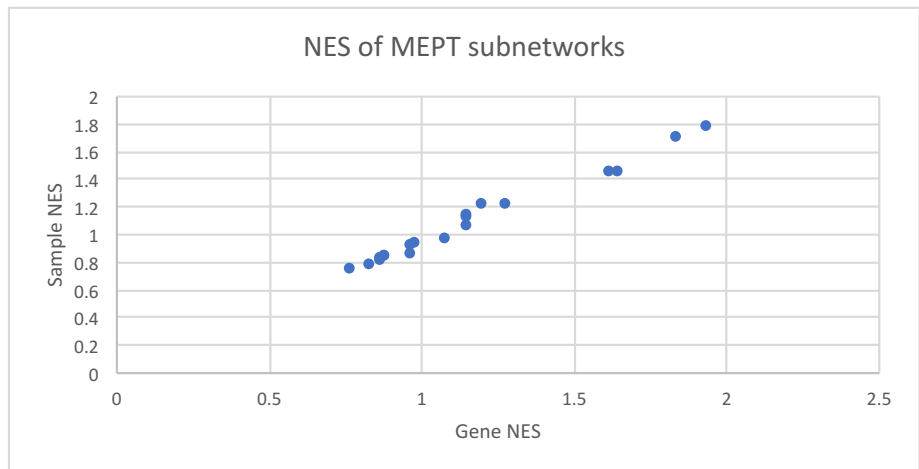

D

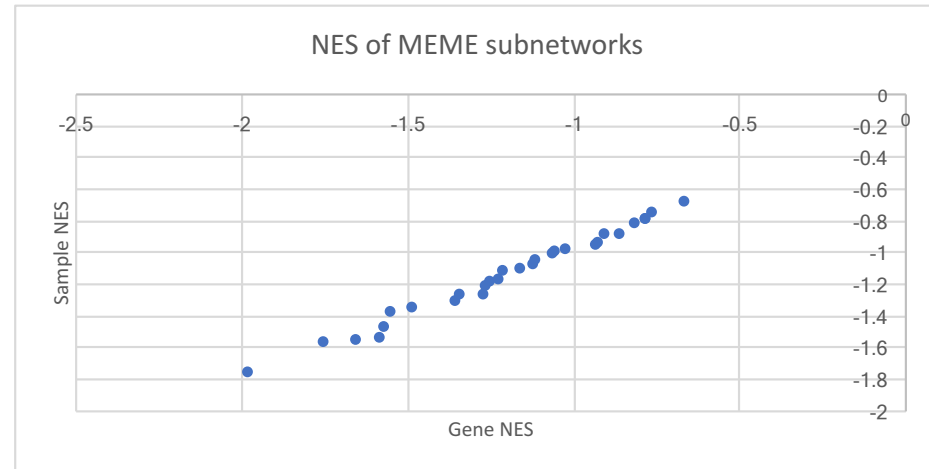

Supplement: S1 Fig — Plots show correlation of NES between gene and sample-based permutation of GSEA of the identified subnetworks. The correlation plots of four types of subnetworks derived according to their initial gene-gene correlations source and enrichment of up-regulated genes in a sample group are shown respectively: (A) subnetworks having primary tumors gene-gene correlations and enriched in primary tumor sample group (PTPT); (B) subnetworks having primary tumors gene-gene correlations and enriched in metastasis sample group (PTME); (C) subnetworks having metastatic gene-gene correlations and enriched in primary tumor sample group (MEPT); (D) subnetworks having metastatic gene-gene correlations and enriched in metastasis sample group (MEME). (PDF) [file pone.0206665.s002.pdf]

A

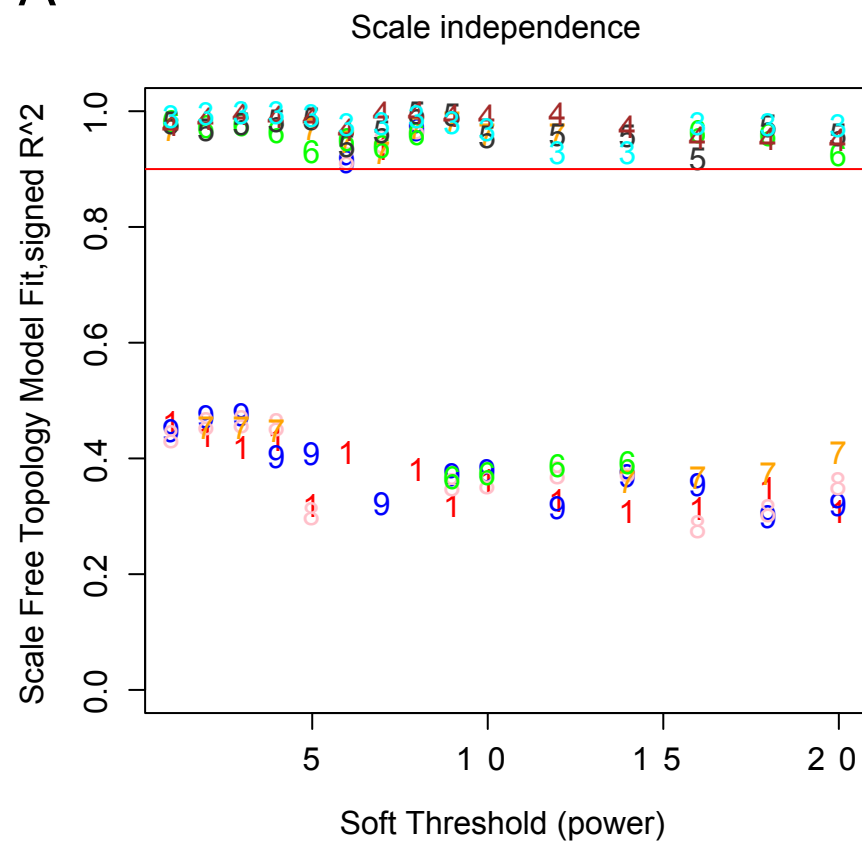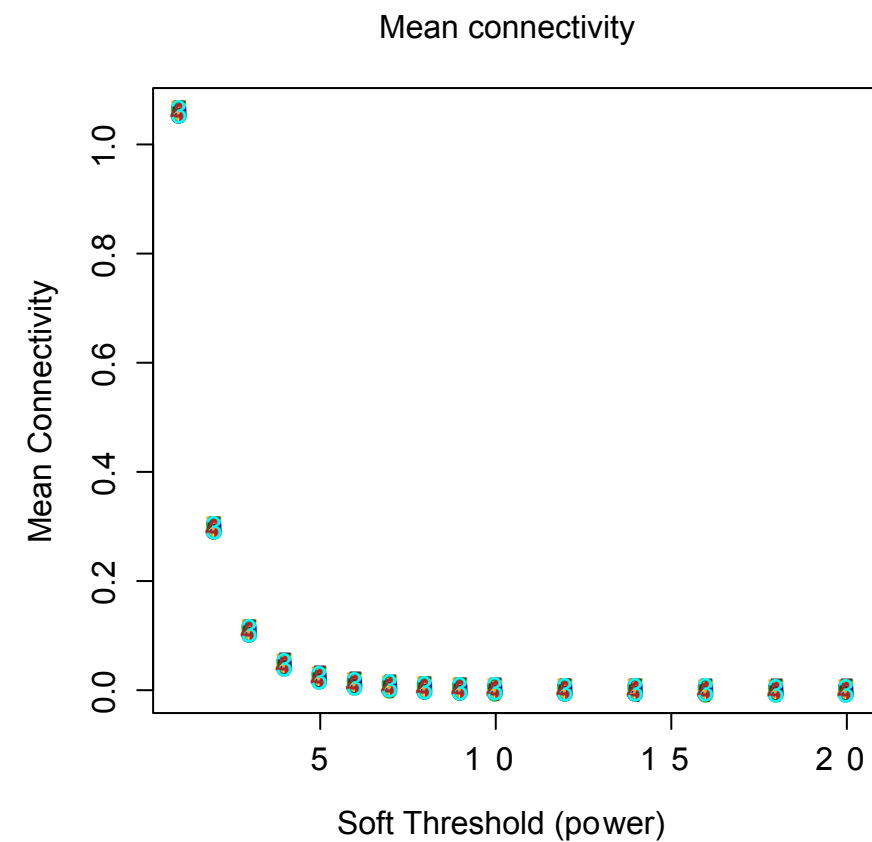

B

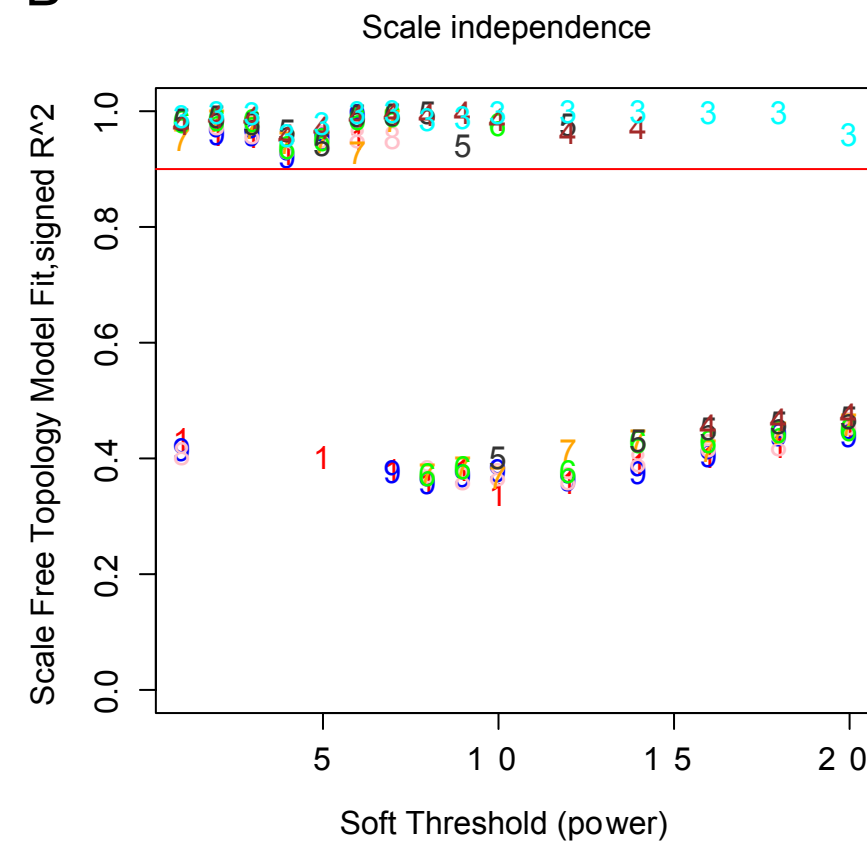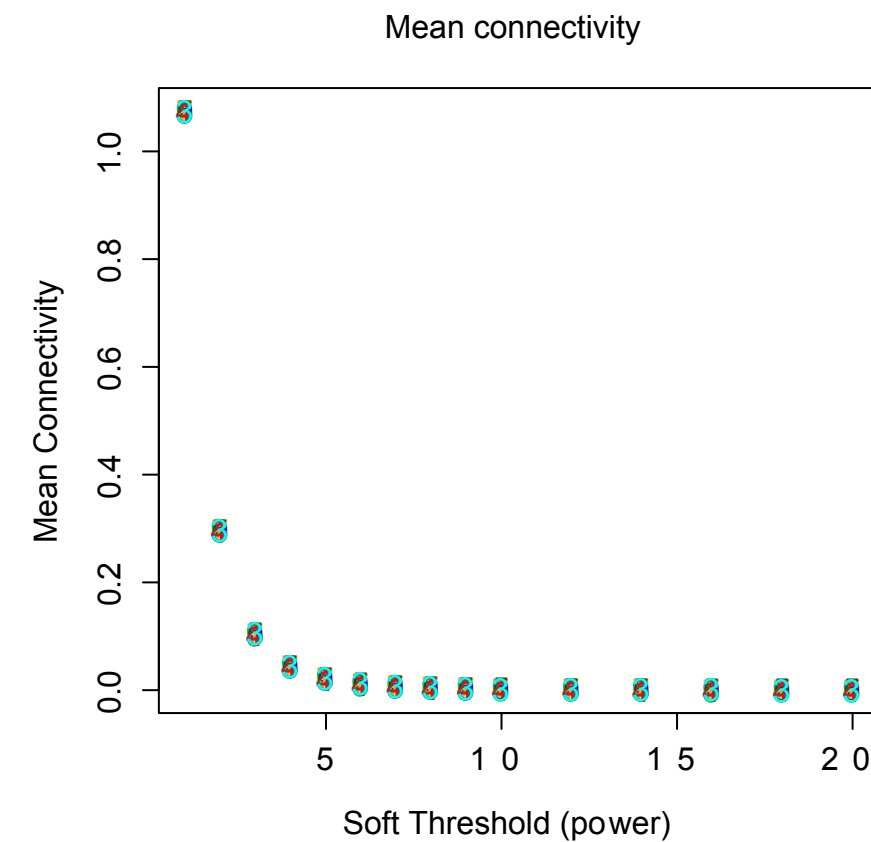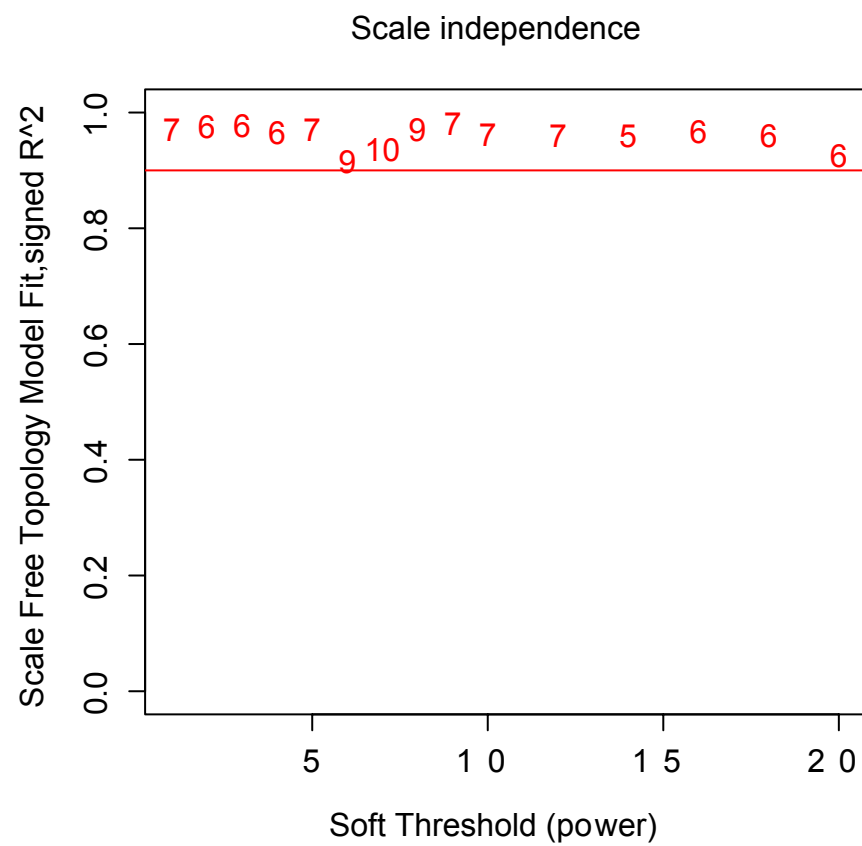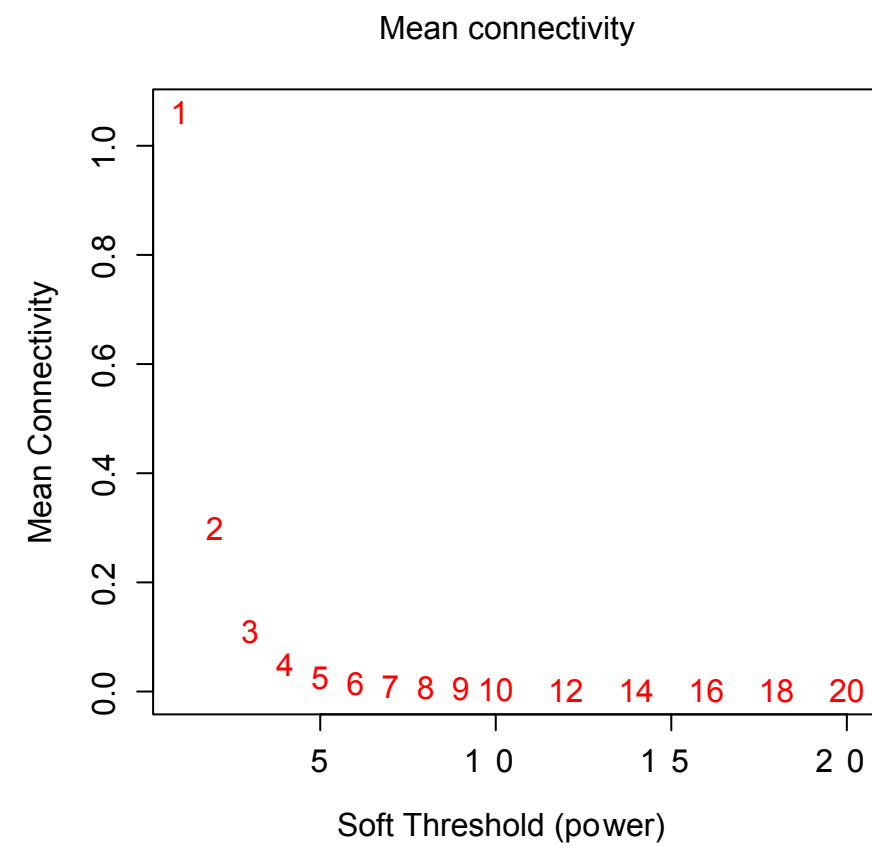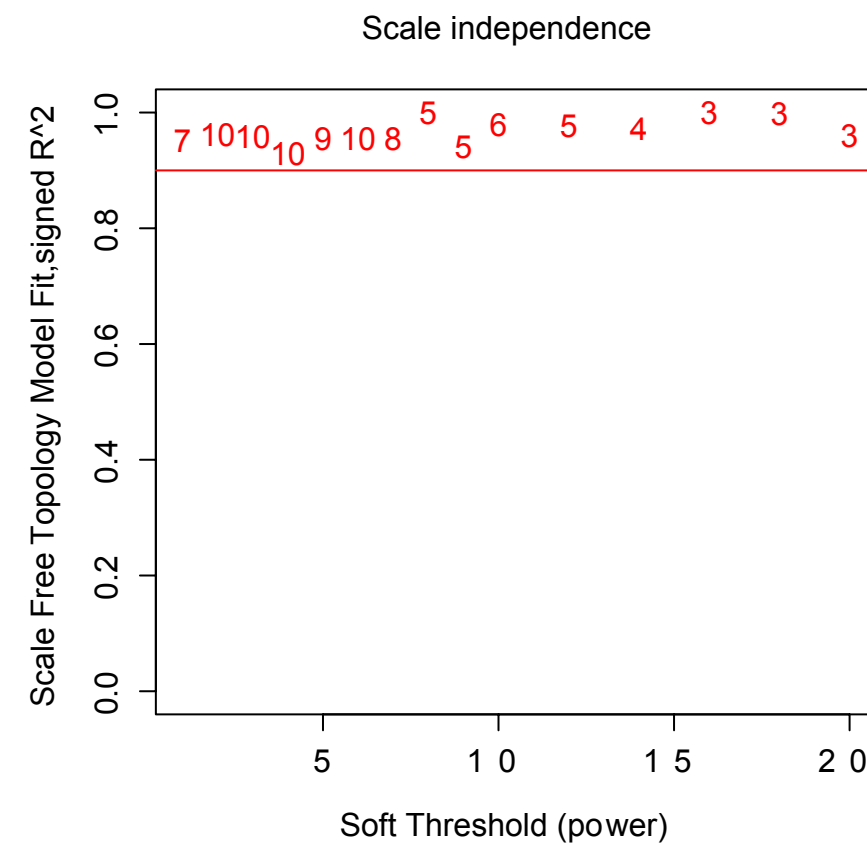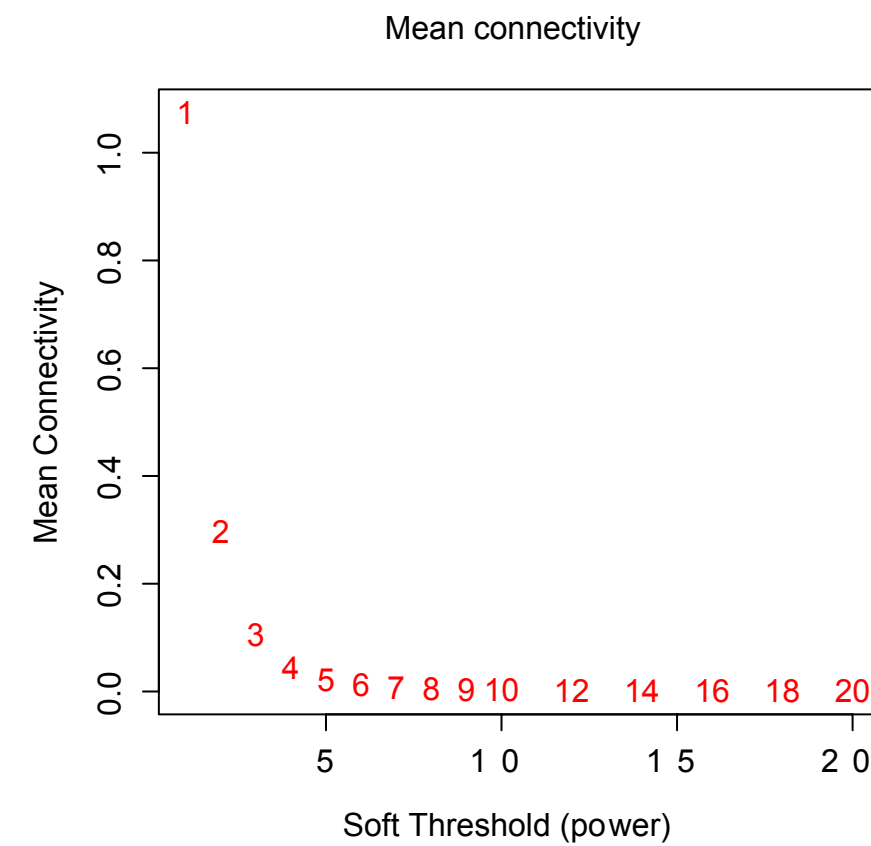

Supplement: S2 Fig — Plots between power parameters of soft threshold vs scale free topology fit and mean connectivity, respectively. The plots of primary tumor (A) and metastasis (B) correlations integrated with PPI are shown, respectively. For each correlation type, plots between power parameters and scale free topology fit (left), and plots between power parameters and mean connectivity (right) are shown. The top panel shows results from different number of bins (from 3 to 10) using for scale free topology fit (1 represent 10 in this plot), while the bottom panel shows results with the optimal number of bin (the biggest bin number while the fit R2 ≥ 0.8). The numbers in the plots represent the numbers of bins except the bottom right plot that the numbers represent power parameters. (PDF) [file pone.0206665.s003.pdf]

A

Network heatmap plot, color genes

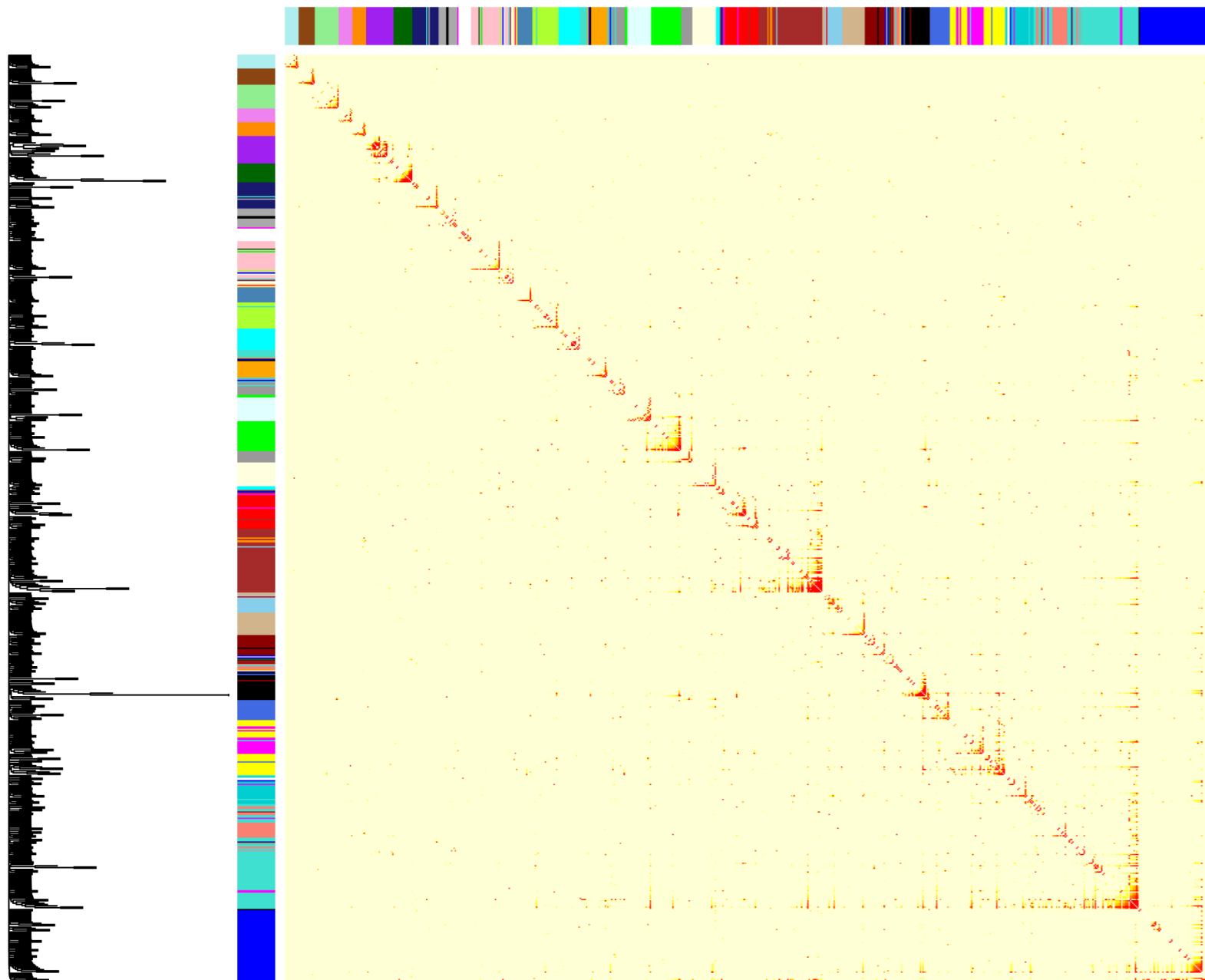

B

Network heatmap plot, color genes

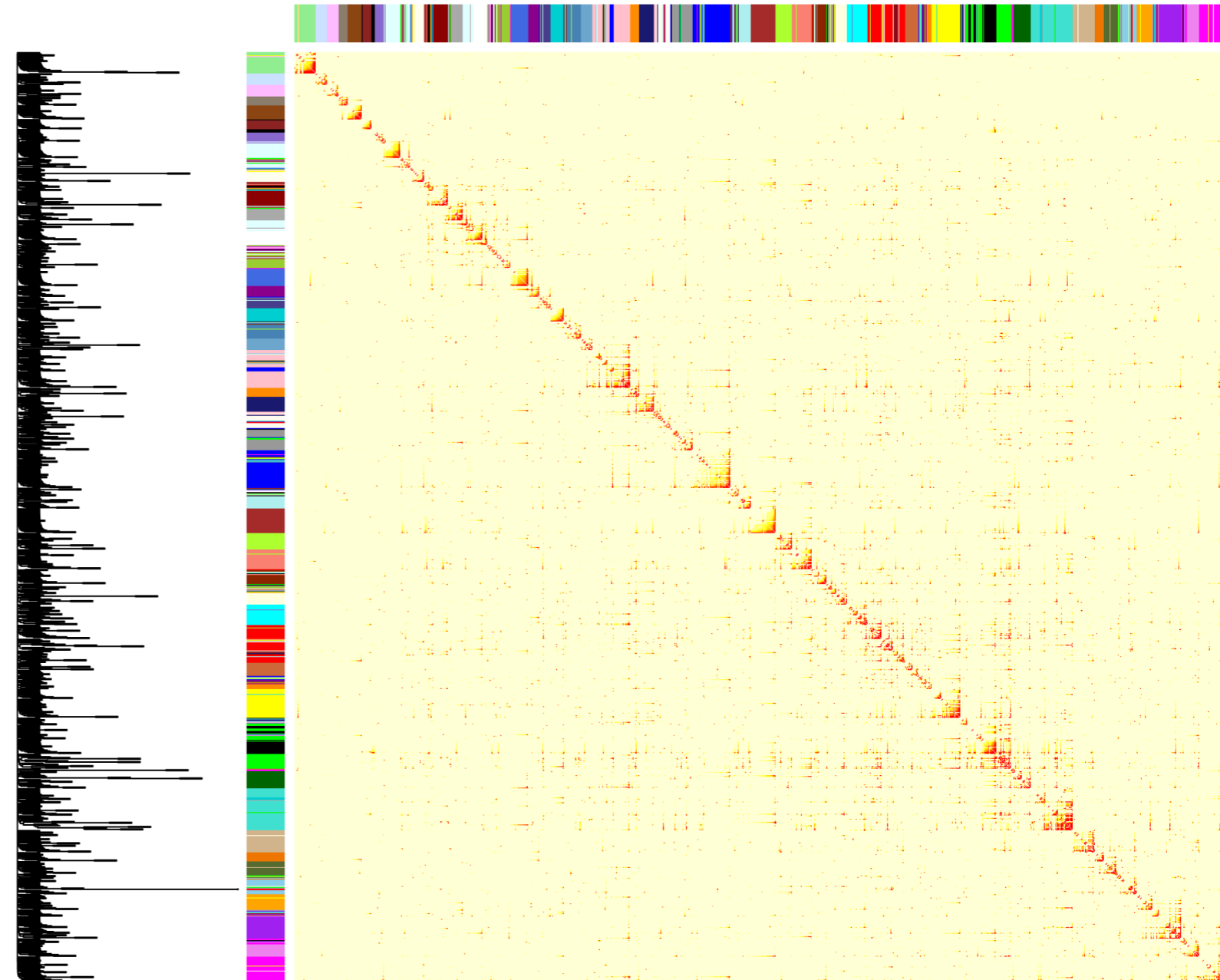

Supplement: S3 Fig — TOM plots of primary tumor (A) and metastatic (B) correlations, respectively. The plots are after integration of the expression correlations and PPI. (PDF) [file pone.0206665.s004.pdf]

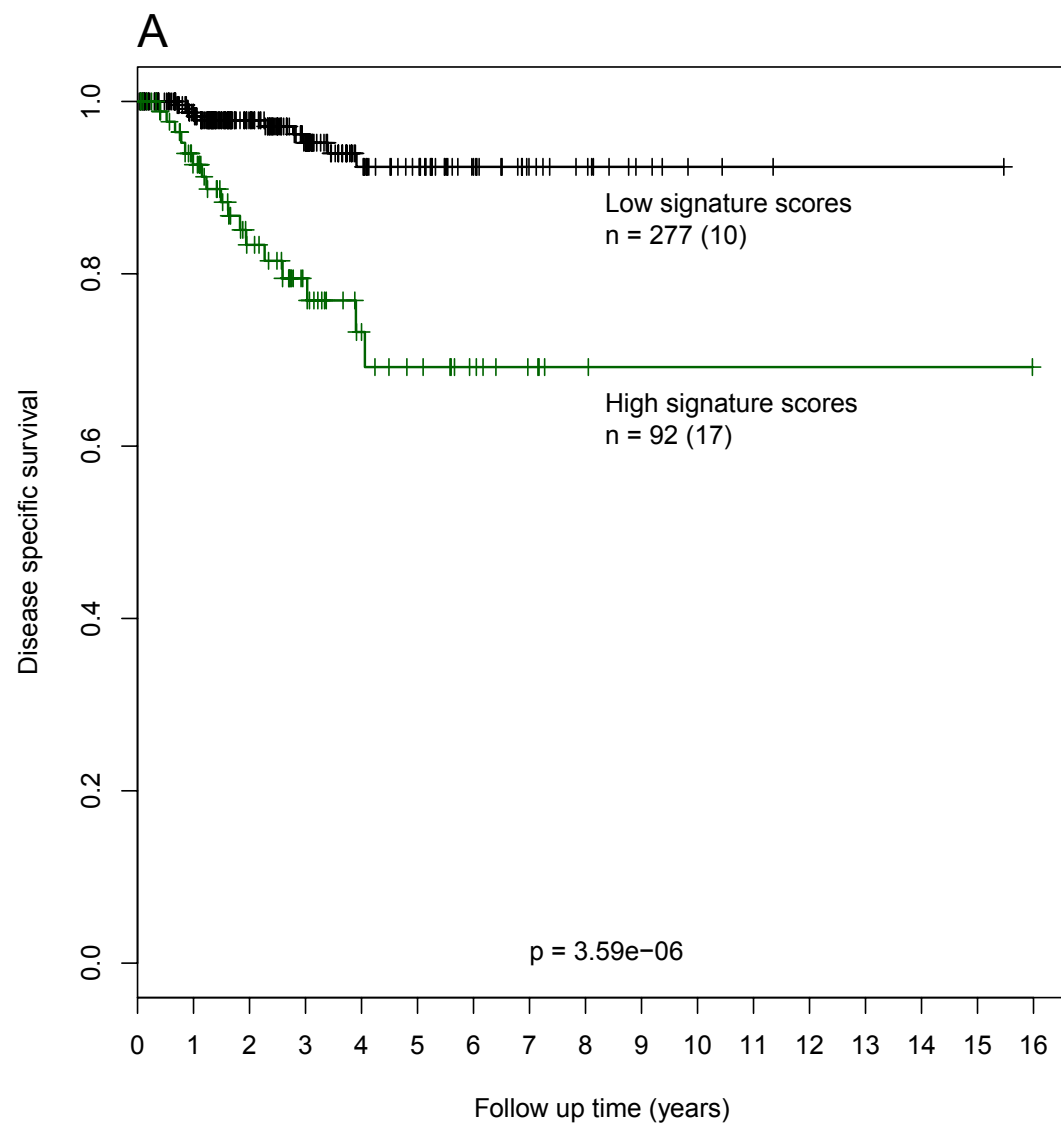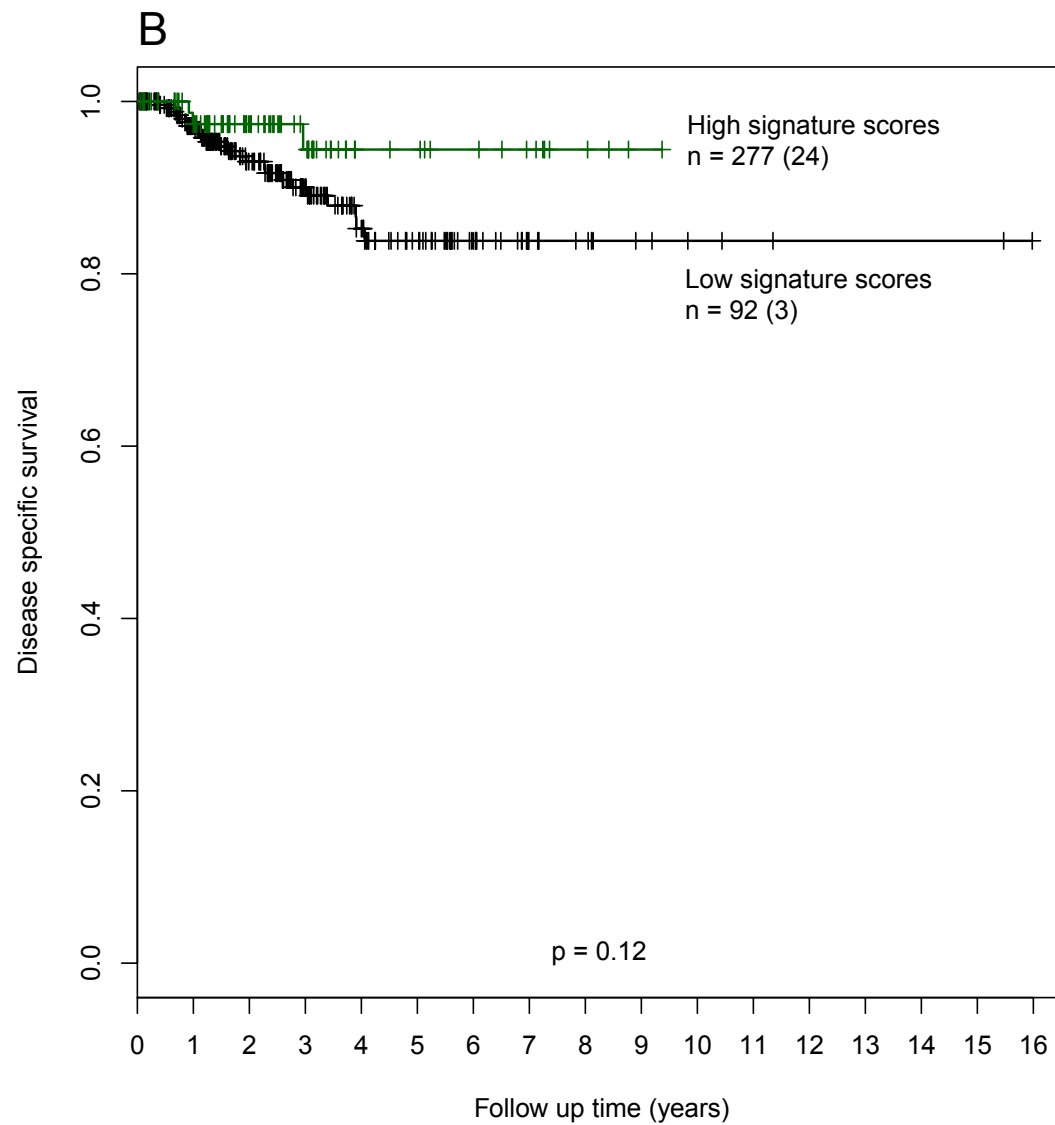

Supplement: S4 Fig — Disease specific survival analyzes according to gene signature scores of the (A) PTME3 (Quantile values of Q1-3 vs Q4 for the patient groups of low and high signature scores, respectively) and (B) MEPT3 (Q1 vs Q2-4 for the groups of low and high signature scores, respectively) subnetworks in TCGA endometrial data. The numbers of patient (n) in each group are displayed with the number of deaths resulting from endometrial carcinoma in parenthesis. (PDF) [file pone.0206665.s005.pdf]
