# Supplementary material for: Identification of highly connected and differentially expressed gene subnetworks in metastasizing endometrial cancer
Source: PLoS One. 2018 Nov 1;13(11):e0206665. doi: 10.1371/journal.pone.0206665 (PMC6211718; doi:10.1371/journal.pone.0206665)
Supplement: S2 Table — The table shows a number of samples used in the Dataset 1, a dataset for subnetwork identification, and Dataset 2, an expanded panel for biological signal investigation of the detected subnetworks. The progression stages from low to high disease aggressiveness are displayed from left to right; complex atypical hyperplasia (CAH), endometrioid primary tumors (ECPT) from grade 1 through 3 (G1, G2 and G3), non-endometrioid primary tumors (NE), and metastatic lesions (ECM), respectively. (PDF) [file pone.0206665.s007.pdf]

S2 Table. Dataset for subnetworks identification (Dataset 1) and for biological signal investigation (Dataset 2). The table shows a number of samples used in the Dataset 1, a dataset for subnetwork identification, and Dataset 2, an expanded panel for biological signal investigation of the detected subnetworks. The progression stages from low to high disease aggressiveness are displayed from left to right; complex atypical hyperplasia (CAH), endometrioid primary tumors (ECPT) from grade 1 through 3 (G1, G2 and G3), non-endometrioid primary tumors (NE), and metastatic lesions (ECM), respectively.

	**CAH**	**ECPT**				**ECM**
		**G1**	**G2**	**G3**	**NE**	
**Dataset 1**	-	Total = 66*				42
		7	14	20	24	
**Dataset 2**	18	Total = 176**				42
		47	52	40	34	

* one case missing data for grade
** three cases missing data for grade
